# The presence of ovarian cysts in a captive Antillean manatee (*Trichechus manatus manatus* L. 1758)

**DOI:** 10.1186/s12917-017-1164-7

**Published:** 2017-08-15

**Authors:** Karolina Goździewska-Harłajczuk, Joanna Klećkowska-Nawrot, Stanisław Dzimira

**Affiliations:** 1 Department of Animal Physiology and Biostructure, Faculty of Veterinary Medicine, Wroclaw University of Environmental and Life Sciences, Kozuchowska 1/3, 51-631 Wroclaw, Poland; 2 Department of Pathology, Faculty of Veterinary Medicine, Wroclaw University of Environmental and Life Sciences, C.K. Norwida 31, 50-375 Wroclaw, Poland

**Keywords:** Case history, Ovarian cysts, Antillean manatee, *Trichechus manatus manatus*

## Abstract

**Background:**

Several pathological changes associated with reproductive systems of marine mammals have been reported in primary literature. However, no such records exist regarding ovarian cysts in the Antillean manatee (*Trichechus manatus manatus* L. 1758).

**Case presentation:**

A nulliparous female Antillean manatee, held in captivity at the Wroclaw Zoological Garden, died in April 2015. The animal was 370 cm long from nose to tail and weighed 670 kg. The width of manatee’s fluke was 80 cm. The *post-mortem* examination of the reproductive system showed the numerous pathological cysts on the external surface of the left and the right ovaries. Morphologically, the cysts had varying diameters and were attached to the ovaries by stalks. Some of the cysts were thin-walled and contained fluid, while several others were solid or contained a semi-solid mass. The structure of the ovaries displayed features of the polycystic ovary syndrome (PCOS). The cysts also exhibited positivity with cytokeratin and vimentin. There were no pathological changes within the uterus, uterine tube and vagina.

**Conclusion:**

Although we were unable to definitively determine the exact source of the ovarian cysts in the studied manatee, we found that one of the causes may be age-related. Our study also revealed that ovarian cysts in the Antillean manatee form both types of corpora lutea (CL).

**Electronic supplementary material:**

The online version of this article (doi:10.1186/s12917-017-1164-7) contains supplementary material, which is available to authorized users.

## Background

The West Indian manatee (*Trichechus manatus* L. 1758), also known as the “sea cow,” the American manatee or lamantine, can be divided into two subspecies: the Florida manatee (*Trichechus manatus latirostris*) and the Antillean or Caribbean manatee (*Trichechus manatus manatus*) [1–5]. According to the classification of the International Union for Conservation of Nature (IUCN, 2008) Red List of Threatened Species, the Antillean manatee is an endangered species [6]. The manatee is also legally protected under the Marine Mammal Protection Act [7]. The Antillean manatees live in freshwater as well as brackish and salty water off the Caribbean coast, off the coast of French Guiana and the Orinoko river [8–11]. The Sirenian reproductive cycle has been widely studied in the Florida manatee [12–15]. Their reproductive activity is seasonal [12]. The length of the reproductive cycle of the manatee may be influenced by environmental factors, such as the water temperature, type of feed and individual characteristics [12–16]. Studies of the Antillean manatees that inhabit fresh water suggest that the availability of vegetation in the high water period and the presence of deep-water areas in the dry period strongly affect their choice of habitat [9]. However, it is still unclear which factors regulate the reproduction cycle in this subspecies of manatee [9]. Moreover, there may be differences in the seasonal reproductive activity between captive and wild manatees [12, 13, 17]. Based on the studies of the Florida manatee, manatees have been classified as polyovular, indicating that several large corpora lutea (CL) are present in its ovaries [18, 19]. Nulliparous sexually mature Florida manatees have an irregular ovarian surface, and there are several Graffian cells (GC) as well as CL or corpora albicantia (CA) present in their ovaries, which suggests that manatees may have a number of oestrus cycles prior to conception [12, 18]. The ovaries in immature (newborn) Amazonian manatees (*Trichechus inunguis*) have a smooth surface without follicles [20]. On the other hand, in the ovulating mature females, they have an irregular surface with numerous GF, located especially in the ovarian cranial pole that have a diameter up to 8.6 mm [20]. In the Amazonian manatee ovaries ruptured follicles (CA) and unruptured follicles (corpora atretica - CAt) were recognized in addition to follicles undergoing atresia [20]. There are two types of CL present in the manatee: CL, one of which results from luteinization of an unruptured follicle, and one which forms from an ovulated follicle [20]. CL develop as a result of luteinization of the remaining granulosa and theca cells. The diameter of multiple CL on the ovaries of the Florida manatee ranges from 4.8 to 7.6 mm [18] or 2 to 9.5 mm [19], while the GF may be two times larger than the CL. The length of the oestrus cycle of the Florida manatee varies from 28 to 42 days [13]. Studies of the CL in mammals revealed the presence of two types of steroidogenic cells: the granular lutein cells (GLC) and the theca lutein cells (TLC) [19, 21], which was confirmed in the CL of the Florida manatee [19]. The correct development of individual ovarian follicles is regulated by endocrine, paracrine and autocrin mechanisms [22]. In mammals, the transforming growth factor β (TGFβ), which can occur as the TGFβ_1_ and TGFβ_2_ subtype, is one of the main intrafollicular regulator proteins [22–24]. A previous study in porcine ovaries showed that TGFβ_1,_ produced by theca cells, affected the growth and differentiation of follicle cells, as well as the stromal reorganization in follicles [22]. Dysfunction of the control of the follicular development may lead to the formation of abnormal follicles (with active secretory activity), which, in effect, may cause dysregulation of the entire reproductive cycle and infertility.

Ovarian cysts are a disorder of the ovaries. They may be functional or pathologic. They can be divided into three groups: follicular cysts (single, multiple, unilateral, bilateral), luteal cysts and CL cysts [35]. Follicular cysts form from follicles, which are not released during ovulation and continue to grow, while luteal cysts form from follicles after ovulation [25]. Follicular cysts contain granular cells, including fragments of the cumulus oophorus. They are thin-walled and filled with fluid. Luteal cysts are derived from follicular cysts whose walls have undergone luteinisation. They are firm and contain a thick wall. CL cysts are a type of functional ovarian cyst and develop from the CL, which does not regress and enlarges after ovulation. Several forms of ovarian cyst may be distinguished based on another classification, including simple serous, endometrial (occurring in the course of endometriosis), dermoid, mucus-filled cysts and cysts containing a solid mass [26]. Ovarian cysts may be benign (resulting from an unruptured follicle), but they may also form a malignant lesion, such as the cystadenoma or cystadenocarcinoma [27–29].

Studies on ovarian cysts are most commonly reported in humans [30, 31], cattle [32–34], pigs [35], horses [36], goats [37], sheep [38], dogs [39, 40], cats [41], guinea pigs [42, 43], mice [44, 45] and rhesus monkeys [46]. There have also been several reports of ovarian cysts in marine mammals, such as dugongs [47] and dolphins [48]. An ovarian tumour and uterine tumour (leiomyoma and carcinoma) were described in a Florida manatee (*Trichechus latistrosis*) [49]. Additionally, an ovarian neoplasm was recognized in the Southern Elephant seal (*Mirounga leonina*) [50], and uterine tumour was found in a grey seal (*Halichoerus grypus*) [51, 52].

To the authors’ knowledge, there exists no description of ovarian cysts in the Antillean manatee in literature. Hence, the aim of this study was the description of a case of ovarian cysts observed during necropsy in an Antillean manatee. This examination could be of interest to those studying marine mammals as well as husbandry specialists and clinicians.

## Case presentation

**Fig. 1:**
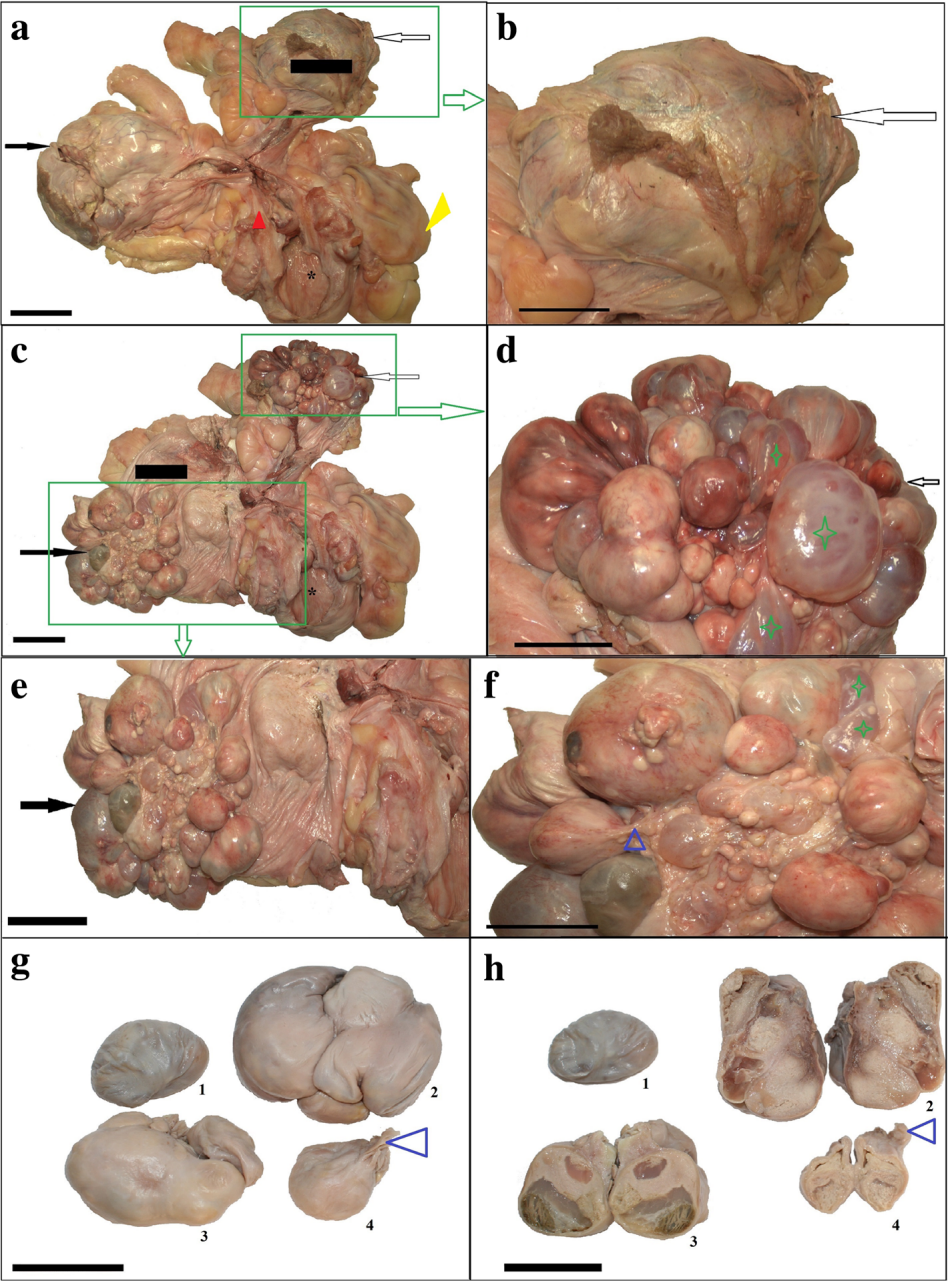
Appearance of the female reproductive organs in the Antillean manatee examined in this study. **a** right ovary (*black arrow*) and left ovary (*white arrow*) with *bursa ovarica* (*black line*), uterine tube, uterus (*red arrow*), vagina (*black asterisk*) and *ligamentum latum uteri* (*yellow arrow*). Scale bar = 10 cm. **b** Left ovary (*white arrow*). Scale bar = 5 cm. **c** Right and left ovaries without *bursa ovarica*. Scale bar = 10 cm. **d** Many ovarian cysts within left ovary. Cysts with fluid (*green asterisks*). Scale bar = 5 cm. **e** Right ovary. Scale bar = 10 cm. **f** The right ovary with many ovarian cysts - magnification of e picture. Cysts with fluid (*green asterisks*), stalk (*blue arrow*). Scale bar = 5 cm **g** Four (1–4) ovarian cysts with different irregular shape. Stalk (*blue arrow*). Scale bar = 5 cm. **h** Cross section of ovarian cysts. 1- cyst with thin wall layer and with fluid inside, 2, 3, 4- cysts with shapeless masses and with small area of fluid or gelatinous substance. Stalk (*blue arrow*). Scale bar = 5 cm

**Fig. 2:**
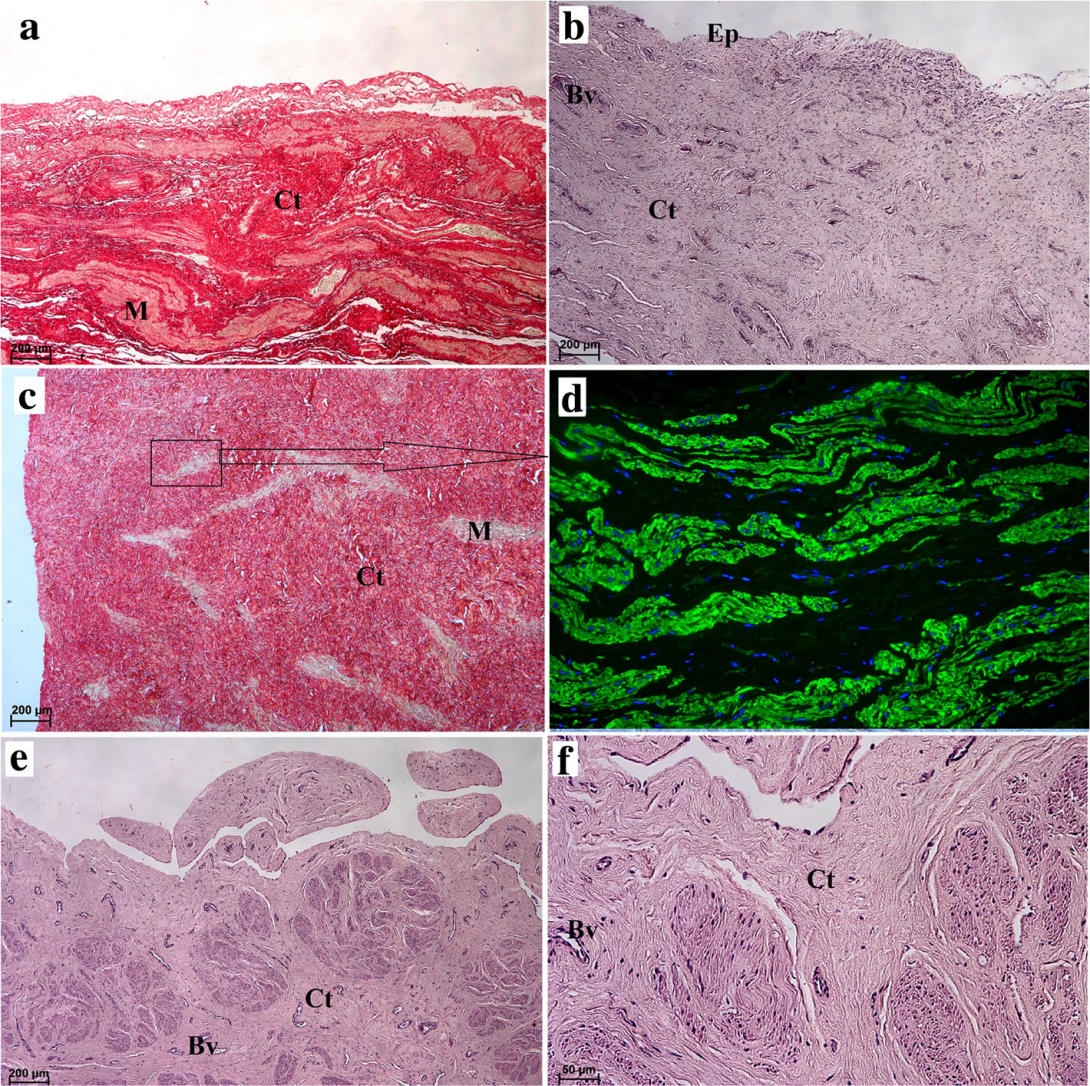
Microscopic structure of the uterus and the uterine tube in the Antillean manatee examined in this study. **a** Picrosirius red staining. Scale bar = 200 μm. **b** H&E staining. Scale bar = 200 μm. **c** Picrosirius red staining. Scale bar = 200 μm. **d** α-actin positive expression. **e**-**f** Uterine tube. H&E staining. Scale bar = 200 μm and 50 μm. *Abbreviations:* Ct: connective tissue; Ep: epithelium; M: muscle; Ac: α actin; Bv: blood vessel

A nulliparous female Antillean manatee was imported from the Tierpark in Berlin (Germany) to the Wroclaw Zoological Garden (Poland) at the end of 2014 year. The animal was 23 years old (born on November 4, 1992 in Nuremberg). Following its arrival at the Wroclaw Zoo, the animal was apathetic, had poor appetite, varying breath amplitudes and an improper posture in water (the animal assumed a vertical position and drifted in the water). Furthermore, it avoided the animal care personnel. According to the information from the Tierpark, the animal had no disease symptoms and did not receive any medication. The animal did not undergo any treatment in the Wroclaw Zoo and died on April 4, 2015. A *post-mortem* study was performed several hours after the animal’s death. The left and right ovaries, as well as the uterine tube, uterus and vagina, were collected for analysis. Morphological, histopathological and immunohistochemical examinations were performed (Additional file 1). The entire reproductive tract (uterus, uterine tube, ovaries, vagina and broad ligaments *ligamentum latum uteri*) weighed approximately 8 kg, and was 55.4 cm long. The vagina measured 15.4 cm from the cervix to the hymen, and had an internal circumference of 6 cm (Fig. 1). The ovarian *bursa ovarica* enclosed each ovary (Fig. 1a-b). The right ovary (together with the ovarian bursa) was 23 cm wide, 24 cm long, 15 cm thick, and weighed 1.8 kg. It contained multiple cysts, which were located on the ovarian surface (Fig. 1c-f). The cysts (10 of which were selected for analysis based on the size) were 65.8 ± 25.6 mm in diameter and were predominantly oval in shape (Fig. 1e-f). Some of them were also irregular. Most of the cysts were attached to the ovary by stalks (Fig. 1f). Some of the cysts were fluid-filled sacs surround by a thin wall, while several cysts appeared as hard, solid masses. In some cysts, there were semi-solid accumulations (Fig. 1g, h). The left ovary (together with the ovarian bursa) was approximately 19 cm wide, 17 cm long, 14 cm thick and weighed 1.4 kg. That ovary was similar in appearance to the right ovary. The fluid-filled cysts (10 selected larger cysts) were 63.8 ± 24.2 mm in diameter and were of different shapes (Fig. 1c-d). Cystic cavities were present in both the right and the left ovaries (Fig. 1c-d). Additionally, the surface lining showed evidence of hematoma around the edge of the cysts, mainly in the left ovary (Fig. 1d) in comparison to the right ovary (Fig. 1f). The microscopic examination revealed the presence of a poorly differentiated epithelium and mucosa of the uterus and uterine tube, suggesting autolytic changes (Fig. 2a-c and e-f). An abundant layer of smooth muscle and connective tissue was detected in specimens from the wall of the uterus and cervix (Fig. 2a-d). In some segments of the cervix, there was a predominance of connective tissue and collagen fibres over smooth muscle cells (Fig. 2c).

**Fig. 3:**
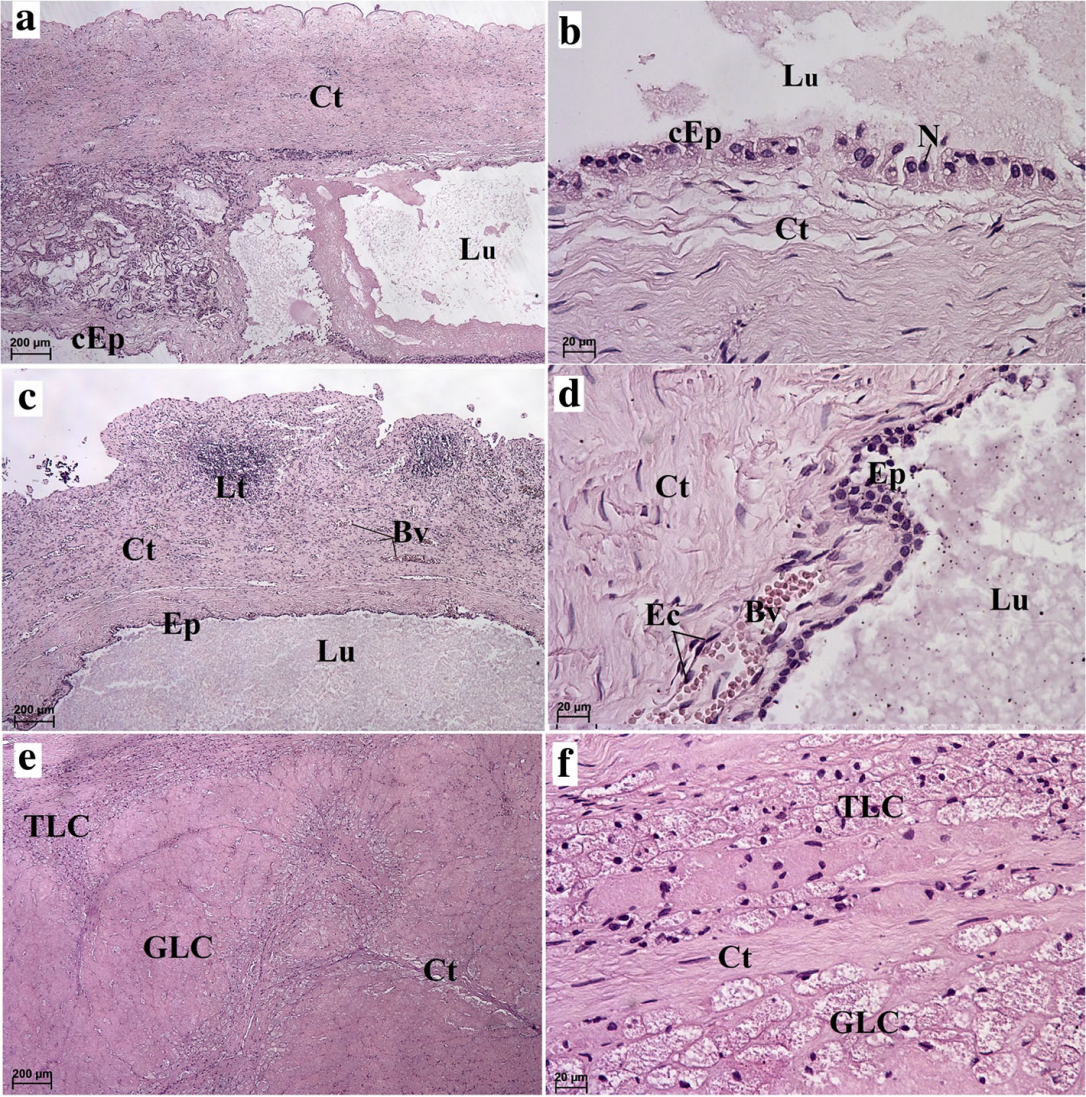
Microscopic appearance of the type of ovarian cysts (I type of CL cysts) in the Antillean manatee examined in this study. **a** H&E staining. Scale bar = 200 μm. **b** H&E staining. Scale bar = 20 μm. **c** H&E staining. Scale bar: 200 μm. **d** H&E staining. Scale bar = 20 μm. **e** H&E staining. Scale bar = 200 μm. **f** H&E staining. Scale bar = 20 μm. *Abbreviations:* Ct: connective tissue; Ec: endothelial cells; Ep: epithelium; cEp: columnar epithelium; Lu: lumen; GLC: granular lutein cells; TLC: theca lutein cells; Bv: blood vessel; N: nucleus

**Fig. 4:**
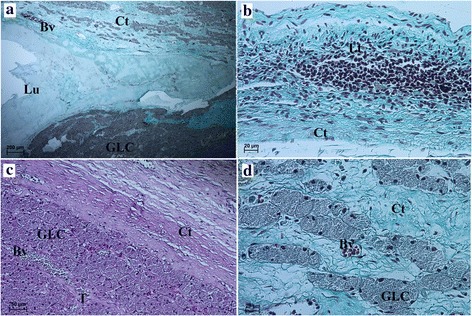
Microscopic appearance of the ovarian cysts (II type of CL cysts) in the Antillean manatee examined in this study. **a** Masson-Goldner trichrome staining. Scale bar = 200 μm. **b** Masson-Goldner trichrome staining. Scale bar = 20 μm. **c** PAS staining. Scale bar = 50 μm. **d** Masson-Goldner trichrome staining. Scale bar = 20 μm. *Abbreviations:* Ct: connective tissue; GLC: granular lutein cells; TLC: theca lutein cells; Lu: lumen (follicular cave); Lt: lymphatic tissue; Bv: blood vessels

**Fig. 5:**
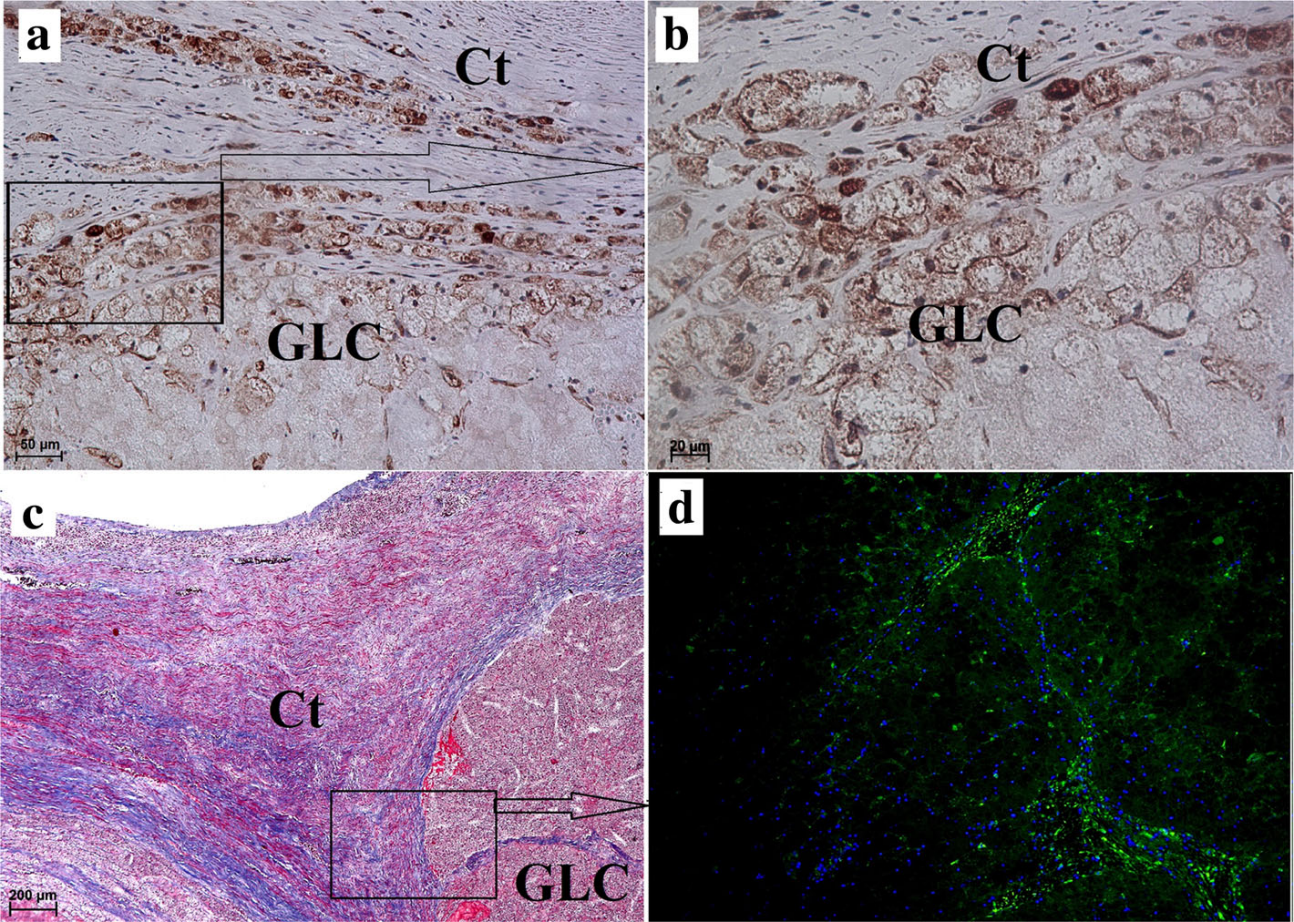
Vimentin expression in corpus luteum cyst in the Antillean manatee examined in this study. **a** Vimentin positive expression. Scale bar = 50 μm. **b** Vimentin. Scale bar = 20 μm. **c** Azan staining. Scale bar = 200 μm. **d** Immunofluorescence. Vimentin positive expression. *Abbreviations:* Ct: connective tissue; GLC: granular lutein cells

Due to the physiological presence of two types of CL in the manatee ovaries, we were able to distinguish type I CL cysts (formed from the CL and caused by the lack of ovulation in the GF) and type II CL cysts (formed from the CL following ovulation in the GF). Small and large ovarian cysts, surrounded by a connective tissue capsule, were found in specimens from the ovarian follicles (Fig. 3). The connective tissue formed septi that extended from the tissue wall into the cyst (Figs. 3e and 5c). There were also structures lined with high columnar epithelium containing a weakly positive eosin fluid (Fig. 3a-b). Next to those structures, we found cysts containing a darker, denser mass lined with a thin flattened epithelium (Fig. 3c-d) or peripheral cells with foamy cytoplasm. Some cysts contained a dark pink, uniform mass (Fig. 3c-d). The most common cysts were lined with connective tissue of varying thickness containing an eosinophilic cytoplasm and a dark blue nucleus (Fig. 3a-d). We also found different kinds of macroscopically visible cysts, which contained numerous cells. There were no amorphous masses with fibrous connective tissue (Figs. 3e, f, and 4c, d). Some of the cystic cavities were divided into two or more sacs (Figs. 3a and 4a). Numerous lymphocytes were found within the connective tissue of the cyst capsules (Figs. 3c and 4b). Smooth muscle cells were visible within the wall of the cysts (Fig. 4b). Numerous fibroblasts with elongated nuclei were visible within the connective tissue of the cysts. (Fig. 3b–f). The fluid that filled some of the cysts stained weakly with hematoxylin and eosin (H&E) or Masson-Goldner trichrome staining. The cystic blood vessels were lined with visibly flattened endothelial cells (Figs. 3d and 4d). The TLC were approximately half the size of the GLC (Figs. 3f and 4d). The TLC and GLC nuclei were oval and were located peripherally (Figs. 3f and 4c, d).

**Fig. 6:**
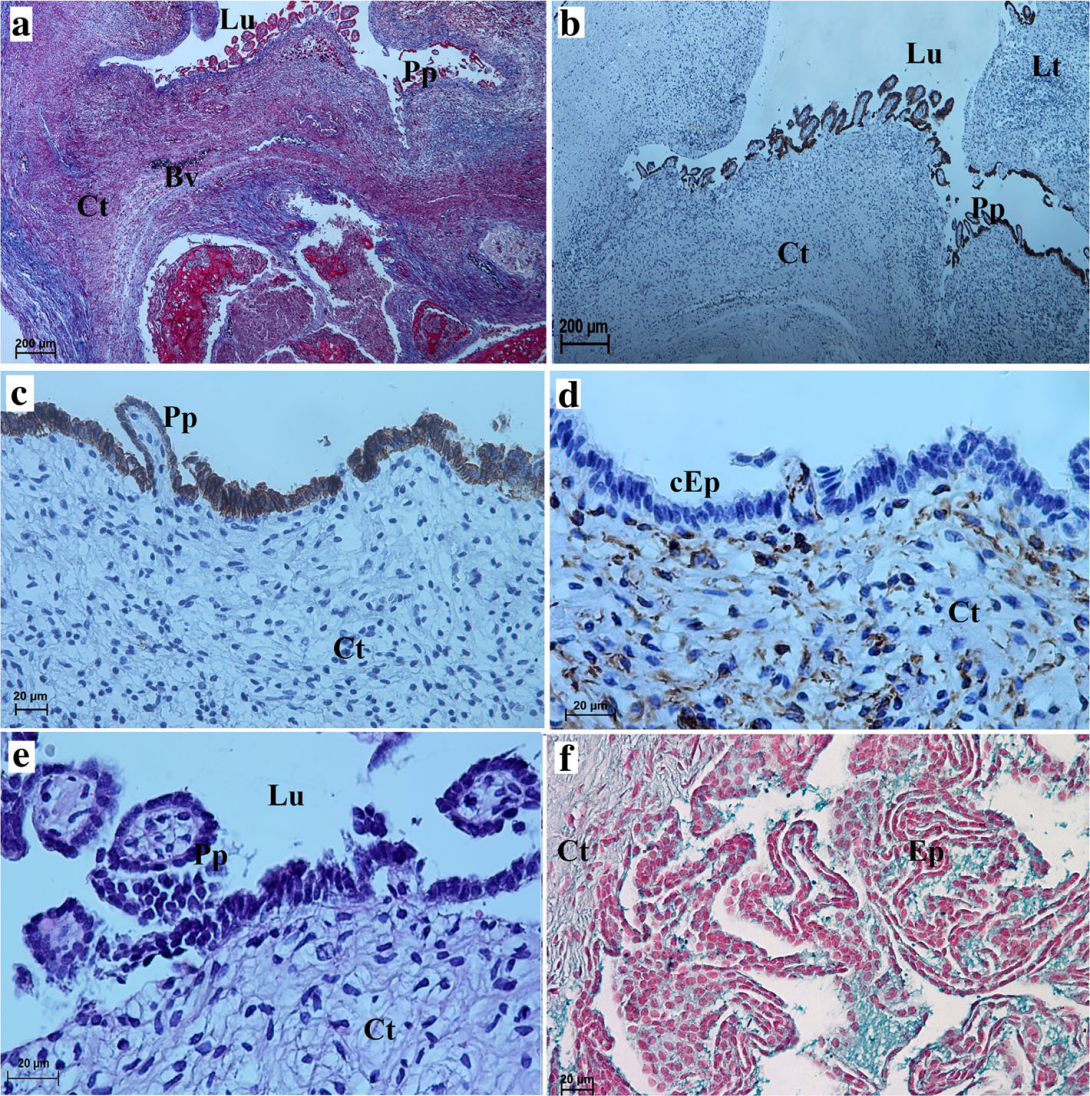
Microscopic structure of ovarian cysts` stalks in the Antillean manatee examined in this study. **a** Azan staining. Scale bar = 200 μm. **b** Cytokeratin positive expression. Scale bar = 200 μm. **c** Cytokeratin positive reaction. Scale bar = 20 μm. **d** Vimentin positive expression. Scale bar = 20 μm. **e** PAS staining. Scale bar = 20 μm. **f** AB pH 2.5 staining. Scale bar = 20 μm. *Abbreviations:* Ct: connective tissue; Ep: epithelium; cEp: columnar epithelium; Pp: papillary structure; Lu: lumen; Lt: lymphatic tissue; Bv: blood vessel

The histological examination of the follicular stalk wall revealed that it had a papillary structure and contained a simple columnar epithelium. However, periodic acid-Schiff (PAS) staining did not show the presence of glycans, glycoconjugates and neutral glycoproteins within the epithelial cells of these papillary structures (Fig. 6a, e). On the other hand, alcian blue pH 2.5 (AB pH 2.5) staining revealed the presence of a small amount of mucins within these papillary structure (Fig. 6f). Immunohistochemistry showed that the epithelia of the cysts expressed cytokeratin (Figs. 6b-c). In the Antillean manatee, vimentin was strongly expressed by the stromal cells of the connective tissue and in ovarian TLCs and GLCs (Figs. 5 and 6d), but it was not found within the epithelium.

## Discussion and Conclusion

Ovarian cysts diagnosed in humans and some animal species can be divided into physiological cysts (functional ovarian cysts) and pathological benign cysts [30–33]. Sometimes, they can transform into neoplastic tumours, such as an ovarian serous or an ovarian mucous cystadenoma or malignant tumours such as mucinous, serous or papillary cystadenocarcinoma [53, 54], as well as teratoma [55]. Neoplastic ovarian cysts can be life-threatening. In our study, the pathological changes in the Antillean manatee ovaries proved to be cysts, previously described in other animal species [47, 48]. This is contrary to the findings of the study on the female Florida manatee (*Trichechus manatus latistrosis*), where neoplastic changes, such as a granulosa cell layer and an ovarian adnexal tumour, were recognized [49]. The lesions found within the reproductive organ of the female manatee examined in this study could have induced reproductive problems, but were not the main cause of the animal’s death. The ovarian cysts in this case report were not malignant and probably grew slowly. Based on the macroscopic images of the cysts in the manatee, they could be divided into simple ones with a thin and smooth wall, and complex ones with a thick connective tissue wall and various intracystic structures. However, due to the presence of two types of CL in the manatee ovaries luteal cysts may form from non-ovulating follicles or from CL after GF ovulation. Based on the histological assessment of the cysts in the studied Antillean manatee, we found that they were of both CL types. Numerous lymphocyte infiltrates were found in the cystic walls, indicating an inflammatory process. The cysts may have caused infertility in the studied female.

Despite improved diagnostic methods, ovarian masses are frequently detected too late. Ultrasonography is a commonly used non-invasive diagnostic technique in medicine and veterinary medicine. However, this technique has limited use in marine mammals due to the presence of large amounts of adipose tissue surrounding internal organs. Therefore, the detection of ovarian cysts in the described manatee was possible only after necropsy. The pathogenesis of the formation of ovarian cysts is well known in humans [30, 31], cattle [32–34], pigs [35], cats [41] and dogs [39, 40]. There is no available data regarding the formation of ovarian cysts in female manatees. To date, the only reported reproductive organ pathologies in the manatee were omphalitis and peritonitis in a young individual [56] and uterine and ovarian neoplasms [49]. Other studies on the reproductive tract in the manatee quantified serum progesterone concentrations in pregnant females [57] and established the morphological features of the mammary glands in the Amazonian manatee [20]. CL cysts have been described in female dolphins [48], and ovarian cysts have been described in the dugong [47]. The ovarian cysts (fluid-filled cysts and adipose cyst) in the dugong were found to be related to age and reproductive activity [47]. Furthermore, a study focusing on Florida manatee females showed that the presence of a reproductive system leiomyoma may depend on the age of the animal [49]. There are several factors apart from age that may significantly affect ovarian lesions in marine mammals.

As in land mammals, the occurrence of ovarian cysts in marine mammals may have a genetic origin. On the other hand, diet, living conditions, stress and age play a crucial role in the development of hormonal imbalance and, subsequently, lead to the formation of cysts or reproductive organ neoplasia. In cattle, the presence of ovarian cysts may cause behavioural disorders [28, 33, 34]. It is unclear whether such behavioural disturbances occur in marine mammals. Studies on dolphins indicate that ovarian cysts may also form as a result of high levels of polichlorinated biphenyls and related xenobiotics (PCB), which reduce pituitary secretion of the luteinizing hormone [48]. It is unlikely that PCB had an impact on the female manatee in this study since she had remained in a zoo since birth and was unlikely to be have been exposed to PCB toxins.

Immunohistochemical studies play a key role in the diagnosis of pathological lesions in the ovaries [58, 59, 60]. Therefore, tissue markers were used and showed an expression of cytokeratin and vimentin in the ovarian cyst wall. Both of these markers are usually used in the diagnosis of ovarian tumours. Vimentin is a cytoskeletal protein component of the type III intermediate filament, and is used as a marker of the epithelial-to-mesenchymal transitions [61]. Cytokeratin is a protein found in the intracytoplasmic area of epithelial tissue. In the manatee, cytokeratin showed a low expression within the granulosa layer and strong expression in the ovarian cyst epithelium and in the columnar epithelium of the papillary structures at the ovarian pedicle. In rats, a strong expression of vimentin and cytokeratin within the granulosa cells of the cystic follicles is associated with cytogenesis [61]. During cytogenesis, there is a change in the expression of structural proteins, which leads to changes in function at a cellular level [61]. The results of the expression of vimentin and cytokeratin in ovarian cysts in the Antillean manatee examined in our study are comparable to those of the rat study [61]. It would be useful to study the expression of chosen markers in unchanged manatee ovarian tissue. This would enable a comparison of the expression of those markers in healthy and pathologically changed tissue. To date, the role of TGFα in the regulation of the proliferation of the cells in the granulosa cell tumor have been described [62]. However, there are no reports of the influence of this regulatory factor on the follicular development in the Antillean manatee ovaries. Our study of ovarian cysts in the Antillean manatee forms the basis for further detailed research into the reproductive system in this subspecies of manatee. The remaining reproductive organs were unaltered.

The study carried out on the Antillean manatee showed the presence of cysts typical for land mammals, but the animal had two types of CL cysts of various origin. The use of new research techniques enables better interpretation of results and facilitates treatment trials in marine mammals, more of which are kept in captivity. Due to the growing environmental threats to marine mammals, understanding their disorders and possible treatment options may increase captive populations.

## Additional file

Additional file 1: Methods of analysis entire reproductive tract of the Antillean manatee. (DOC 26 kb)

## Abbreviations

Ac: α actin
Bv: Blood vessel
CA: Corpus albicans
CAt: Corpus atreticus
cEp: Columnar epithelium
CL: Corpus luteum
Ct: Connective tissue
Ec: Endothelial cells
Ep: Epithelium
GF: Graffian follicle
GLC: Granular lutein cells
H&E: Hematoxylin and eosin staining
IUNC: International Union for Conservation of Nature
KNOW: Leading National Research Center
Lt: Lymphatic tissue
Lu: Lumen
M: Muscle
N: Nucleus
PAS: Periodic acid Schiff staining
PCB: Polichlorinated biphenyls and related xenobiotics
PCOS: Polycystic ovary syndrome
pH 2.5 AB: pH 2.5 alcian blue staining
Pp: Papillary structure
TGFβ: Transforming growth factors β
TGFβ_1_: Transforming growth factor β_1_
TGFβ_2_: Transforming growth factor β_2_
TLC: Theca lutein cells
