# Supplementary material for: The presence of ovarian cysts in a captive Antillean manatee (Trichechus manatus manatus L. 1758)
Source: BMC Vet Res. 2017 Aug 15;13:240. doi: 10.1186/s12917-017-1164-7 (PMC5558751; doi:10.1186/s12917-017-1164-7)
Supplement: Additional file 1: — Methods of analysis entire reproductive tract of the Antillean manatee. (DOC 26 kb) [file 12917_2017_1164_MOESM1_ESM.doc]

**Methods**

Morphological and morphometric measurements of the above mentioned organs were taken using an electronic slide calliper with an accuracy of up to 0.01 mm (Digitronic Caliper, Moore & Wright). The organs were weighed using an electronic scale (AXIS B15 M, Gdańsk, Poland). The terminology used in the manuscript is in accordance with the prevailing veterinary nomenclature (*Nomina Anatomica Veterinaria,* 2012). Histopathological and immunohistochemical examination were performed.

*Histological analysis*

The studied organs were directly fixed in 4% buffered formaldehyde for two weeks, and washed in running water for 24 hours, then processed in an ETP vacuum tissue processor (RVG3, INTELSINT, Italy). They were embedded in paraffin and cut using a Slide 2003 (Pfm A.g., Germany) sliding microtome into 3-4 µm sections. The samples were then stained with H&E, Masson-Goldner trichrome, Azan trichrome and picrosirius red to evaluate the structure of the tissues. The PAS method was used to visualise glycans, glycoconjugates and neutral glycoproteins, while the AB pH 2.5 method was applied to detect acid sialylated glycosaminoglycans. A Zeiss Axio Scope A1 light microscope (Carl Zeiss, Jena, Germany) was used to examine the slides.

*Immunohistochemical analysis*

The sections for immunohistochemistry were deparaffinised and inactivated in 3% hydrogen peroxide for 10 minutes at 23^o^C temperature. The samples were incubated with a primary antibody (for 60 min) and then with a secondary antibody (for 30 min). The sections were stained immunohistochemically using the following antibodies: cytokeratin (Abcam, cat. No. ab961), vimentin (Santa Cruz, cat. No. sc73259) and alfa-smooth muscle actin (Abcam, cat. No. ab5694). This examination was carried out in the Institute of Animal Physiology and Genetics, v.v.i., Academy of Sciences of the Czech Republic in Brno.
